# Personal and joint liability of partner in limited liability company in UAE federal corporate law No. 32 of 2021

**DOI:** 10.1016/j.heliyon.2022.e10322

**Published:** 2022-08-24

**Authors:** Nazzal Kisswani, Ahmad Farah

**Affiliations:** Associates Professors of Commercial Law, College of Law, University of Sharjah, United Arab Emirates

**Keywords:** Limited liability, Personal liability, Joint liability

## Abstract

The limited liability company (LLC) is regarded as a significant contributor to the national economy. Many people use it because they believe their liability is limited to the amount of their capital share. The UAE lawmaker recognized this in the Commercial Companies Law No. 32 of 2021, when it identified cases in which the partners’ liability for debts and obligations becomes personal and joint in the event of violation of the law or fraud by the partners or managers of the company. As a result, the capital is not the only guarantee for the creditors of the company. This study aims to analyze the legal texts and elucidate the cases in which the partners' liability is regarded personal and joint.

## 1 Introduction

The limited liability company is one of the most common and traded commercial companies in the United Arab Emirates, as it is the preferred model for people when establishing commercial projects. The reason for this company’s preference is that it responds to businessmen’s desires for establishing medium and small enterprises without assuming absolute liability for complex foundational procedures. Furthermore, this company plays a crucial role in the stability of the corporate sector in the state, as it aids in the continuation of existing companies if something happens to their owners that prevents them from continuing, such as the death of one of them. For example, rather than dissolving the company, the heirs can continue to operate it.

The limited liability company is a company of mixed nature^1^, as it is considered to be somewhere between companies of persons^2^ and associations of capital^3^. In the sense that it has mixed characteristics, some of its provisions serve personal considerations, such as the number of partners and the associations of capital, where the partners have limited liability for the company’s debts.

As a result, the liability of the partners in the limited liability company is limited to the amount of the shares they provided in the capital, provided the partners abide by the provisions of the Companies Law. In the event that the partner or partners do not abide by the provisions stipulated by the law, the partners’ liability shifts from limited liability to personal and joint liability.

The UAE lawmaker, in Chapter Three of Federal Law No. 32 of 2021 concerning Commercial Companies^4^, has regulated the provisions of the Limited Liability Company. With the issuance of this law, all that contradicts or conflicts with this law has been canceled, in addition to the cancellation of Federal Law No. - 8 of 1984 in the matter of commercial companies and the laws amending it. According to Article 71 of the new law on limited liability companies, a single person, a natural or legal citizen, may establish and own a limited liability company, and the owner of the company's capital is not liable for its obligations except to the amount of the incoming capital in a manner that does not contradict its nature. The provisions of the limited liability company mentioned in this law shall apply to him in contradiction with its nature.

In this context, this research seeks to analyze the legal texts of the limited liability company and indicate the cases in which the liability of the partners is not defined, but rather it has become a personal and joint liability of those who attempt to cheat or violate the law, as the lawmaker no longer protects the partners only, but also seeks to protect those dealing with them, with the aim of finding a balance between the interests of partners and the interests of others in this type of company.

## 2 Overview of the limited liability company in the UAE law

The limited liability company first appeared in Germany in 1892. It spread throughout Europe after flourishing in Germany. Austria was the second country to organize this type of company in 1906, followed by England in 1908, and France for the first time in 1925, when it moved to it through the French provinces occupied by Germany^5^.

It can be said that the designation of the limited liability company is a translation of the designation of this company in the French. However, some argue that the Arabic name is incorrect because the partners, not the company, are the ones whose liability is defined^6^. In the United Arab Emirates, the first appearance of this type of company was in 1984 when the Commercial Companies Law No. 8 of 1984 was issued. Then came the new Companies Law No.2 of 2015, which repealed the Federal Law No. 8 of 1989 on commercial companies, as amended^7^. Then came the new Companies Law No.32 of 2021, the new Law repeals the previous commercial companies, which recently came into force on 2 January 2022.

The new law seeks to keep up with the tremendous development in commercial relations, economic openness and global changes. Article 2 of the UAE Commercial Companies Law stipulates that “This law aims to contribute to the development of the business environment, the capabilities of the state and its economic position by organizing companies in accordance with global variables, especially those related to the regulation of rules of governance, protecting the rights of shareholders and partners, supporting the flow of foreign investment and promoting corporate social liability”. The new law also broadened the scope of the law's application to include foreign companies mentioned in the law, as well as foreign companies for which the UAE is a center for performing any activity or establishing a branch or office in it^8^.

The UAE lawmaker defined a limited liability company in Article 71 (1) as “a company in which the number of partners is not less than two and no more than fifty partners, and each of them is only asked to the extent of his share in the capital.” It is clear from this definition that the lawmaker focused on some of the characteristics that distinguish this company in terms of the number of partners in it and the nature of the liability of the partners, provided that its other characteristics that are not mentioned in this text are benefited from other legal texts that regulate the limited liability company. Among its most important characteristics are:

**-** Company name: Article 72 of the UAE Commercial Companies Law stipulates that the company may have a name derived from the name of one or more partners. It may also have a trade name derived from the purpose of its formation. The company's name may not be contrary to the state's public order and morals, or may it be registered under a name that has already been registered or that would cause confusion and ambiguity^9^. It is noted that allowing this company to use a name that includes one or more of the partners' names may cause confusion about the nature of the partners' liability in it. However, we can see that the lawmaker wanted to emphasize that the partner's liability is not always limited. Rather, the liability may become jointly and may extend to the company's own funds.

The lawmaker did not neglect the possibility of confusing the matter with dealers with the company, as they may believe it is another type of commercial company stipulated in Article 9 (1) of the UAE Commercial Companies Law^10^, so he decided the necessity of adding the phrase with limited liability to the company’s name, and it can be referred to briefly (LLC). This is in addition to adding this phrase to the company's papers, bills and contracts^11^.

**-** The number of partners in the company: Article 71 of the UAE Commercial Companies Law stipulates that two partners should establish this company, but the lawmaker has permitted one person to establish or own this company, and therefore it is permissible, as an exception from the original, that the number of partners in this company is less than two^12^.

The lack of tradability of shares: The limited liability company may not prove that the partners’ shares are in negotiable instruments, as Article 31 of the Commercial Companies Law stipulates that only the joint-stock company may issue shares, bonds, or negotiable instruments. Thus, a limited liability company is similar to a person’s company in that the partners own shares, whereas in a joint-stock company the partners are shareholders. Article 76 (2) specifies that the shares of the partners are in cash or kind, and the partners are obligated to fulfill the shares in full, and as a result, the partner's share may not be a work share^13^.

- Prohibition of resorting to public subscription^14^: A limited liability company is prohibited from resorting to public subscription for its shares to form its capital or increase it, and it is also not permitted for it to issue negotiable securities because this is limited to joint-stock companies, which is confirmed by Article 31 of the UAE Commercial Companies Law that “No any person other than the joint-stock company may issue shares, bonds, or negotiable instruments”.
- Prohibition of a limited liability company from practicing some commercial activities: The lawmaker restricted some activities to joint-stock companies only. Article 11(3) of the UAE Commercial Companies Law stipulates that “Only Public Joint Stock Companies may conduct banking and insurance activities. Only Joint Stock Companies may invest money for the account of third parties”. According to the preceding text, the lawmaker decided to define the field of activities that the limited liability company can engage in because of the nature of the limited liability company, which is dependent on a specific capital and the specific liability of the partners^15^.

## 3 Limited liability of the partner in the limited liability company

The limited liability company distinguishes itself by limiting a partner’s liability to the amount of his share of the capital, and this determination serves as the basis for naming this company^16^. The limited liability of the partners means that each partner is only bound by the value of his share in the capital, and it is not permissible to demand more than that. Therefore, the guarantee of its creditors is limited to its financial liabilities without the liabilities of the partners in it. The liability of others for its debts is determined by the amount of each of their share in its capital in an absolute determination that applies to their relationship with each other and their relationship with others^17^. As a result, the company's creditors have no guarantee other than the company's capital declared in the articles of incorporation, with no financial liability of the partners due to their independence from one another. If the company's debts exceed its capital, there is no guarantee for the creditors of the limited liability company except for the financial liability of the company, and they have no right to collect their debts from the partners' private funds^18^.

According to the opinion, the partners' limited liability is based on the concept of allotment liability, according to which the partner deducts a portion of his financial liability to form a commercial project in which his liability to others is limited to the extent of only this portion of the company's capital^19^.

Some legislation in connection with allotment liability have found a suitable legal basis for determining the liability of the commercial enterprise, so that we have two liabilities, one of which is personal and the other for the project, which leads to limiting the liability to the partners in the limited liability company^20^. Another aspect of the jurisprudence sees that the corporate personality of the company gives it a financial liability that is independent of the funds of the partners in the company, as the share provided by the partner comes out of his financial liability and enters into the financial liability of the company and becomes the property of the company, so that the company has the right to dispose of the share in the way it deems appropriate^21^. While some believe that the harmonization between the two main bodies of the legal person and the liability for the allotment and reconciliation between them provides a rationale for determining the liability of the partner in the limited liability company, and it obviates the creation of a new legal framework. The privatized financial liability is attributed to the legal person represented by the limited liability company, and the company is liable for the project's liabilities and debts. Similarly, the allocation of a financial liability by the partners in the company separates his other funds from bearing the obligations of the project, so it is not referred to under the guise that this company is not part of the partners' financial liability^22^.

It is not permissible to question partners after they have fulfilled their shares in full upon incorporation of the company without exceeding the estimated value of in-kind shares. Creditors are not required to make any claim related to the company's debts^23^. This principle applies even if the commercial license of the company has not been renewed, as renewing the license is a regulatory procedure that does not result in its failure to change the legal form of the company^24^.

Although the limited liability company is the most established in the United Arab Emirates in recent years, the company faces a number of issues as a result of the limited liability of the partners.

**Figure 1:**
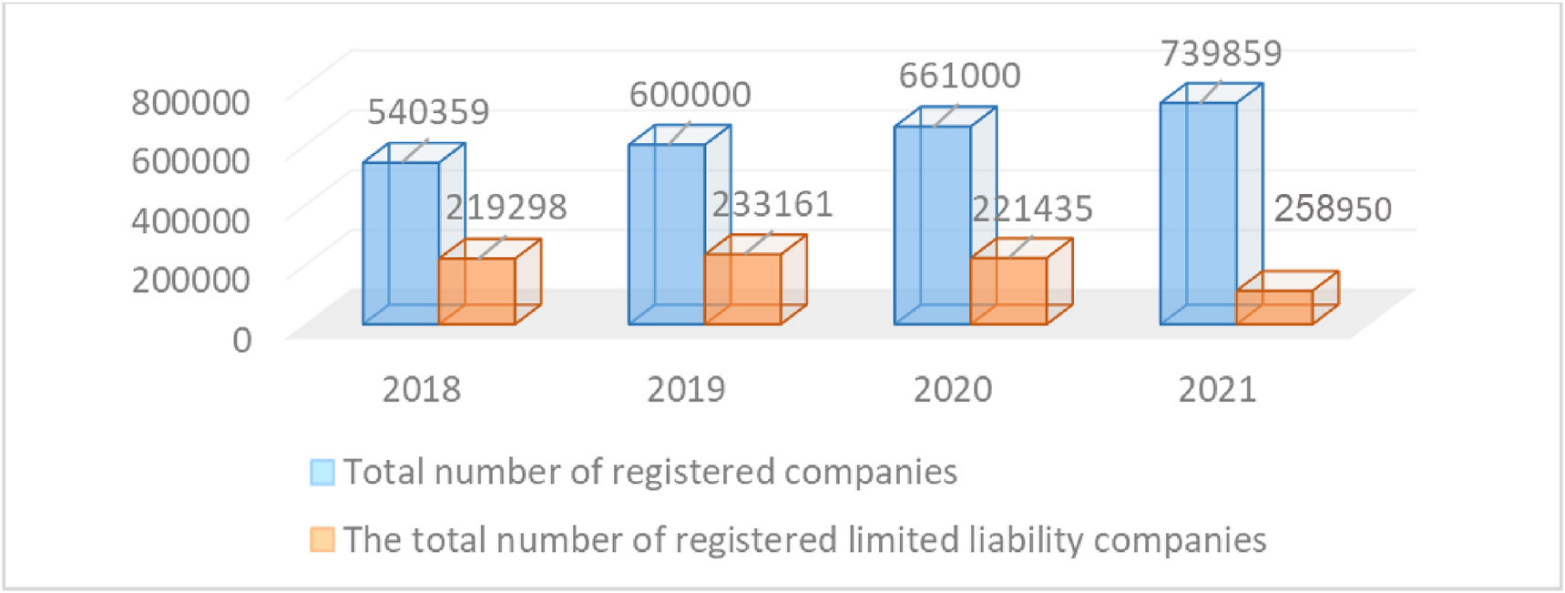
Number of limited lability company in UAE. (2018: https://al-ain.com/article/company-licences 2019: https://www.alkhaleej.ae/2019-09. 2020: https://www.alroeya.com/117-81/2149836-10 2021: https://www.alarabiya.net/aswaq/economy/2021/01/28).

Figure 1 illustrates the increased number of the registered companies and the number of registered limited liability companies in the UAE.

**Figure 2:**
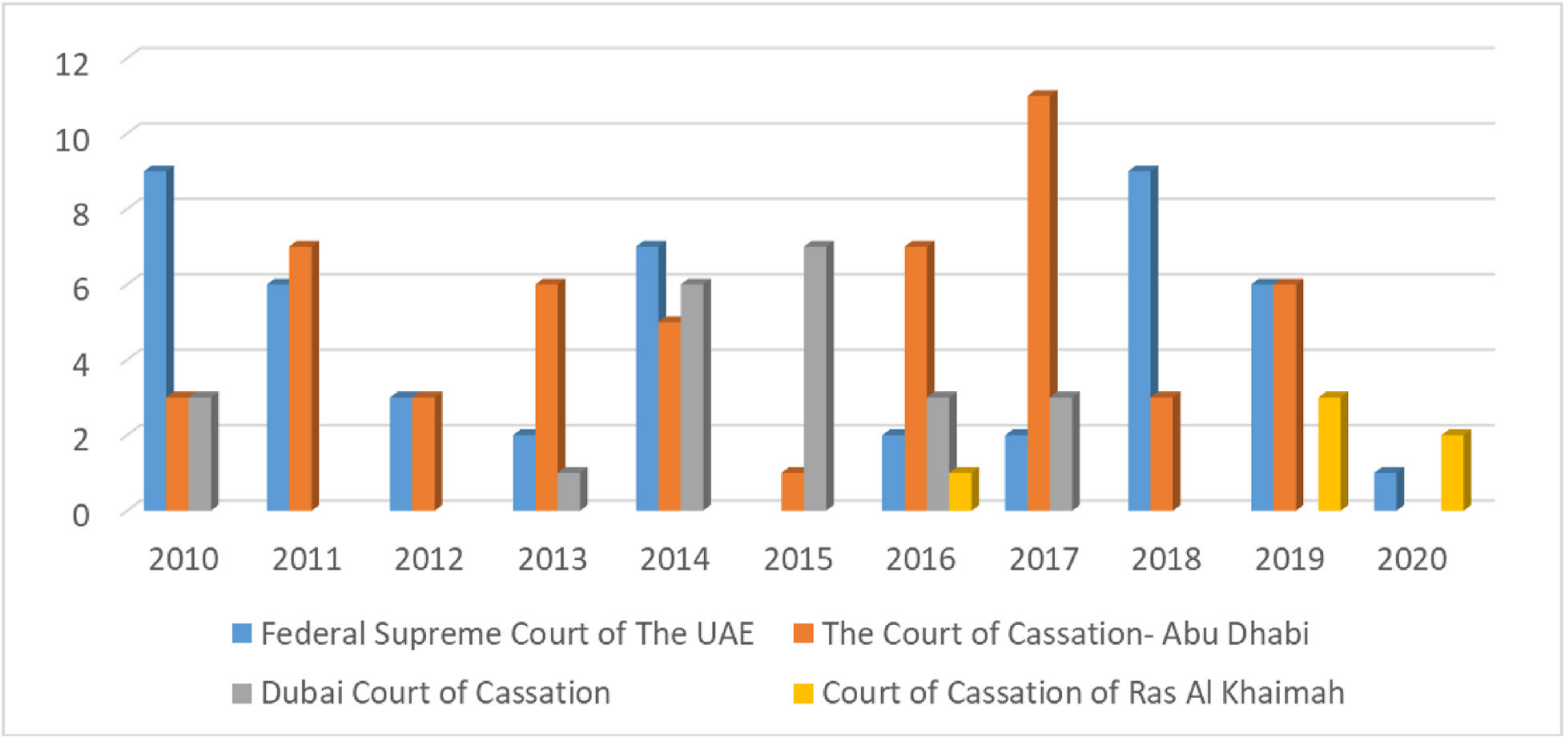
Number of issues (east law data base).

Figure 2 Illustrates the number of court rulings regarding the companies’ violations between 2010 and 2020 at 10-years Intervals. For the Federal Supreme Court of the UAE issued 9 judgments in 2010 and 2018. The same Court issued 7 judgments in 2014, and 6 judgments in 2011 and 2019 (Figure 2).

The Abu Dhabi Court of Cassation swept the largest number of judgments in 2017, when it issued 11 judgments, and issued a 7 judgments in 2011 and 2016 (Figure 2). It shows also that 6 judgments in 2013 and 2019. In addition, Figure 2 shows 3 judgments in 2010, 2012, and 2018. Only a single judgment in 2015. While Dubai Court of Cassation issued 7 judgments in 2015 and 6 judgments in 2014. Also 3 judgments in 2010, 2016, and 2017. As for Court of Cassation of Ras Al Khaimah had issued 3 judgments in 2019, and it was the only court issued a 2 judgments in 2020, indeed only a single judgment in 2016 (Figure 2).

**The Previous figure is distributed between the following:**

- The liability of partners and managers towards third parties for acts of fraud.
- Limited and joint liability of the company's partners and managers.
- Issuance of checks without balance.
- Dispute over the use of trademarks and geographical indications.
- The criminal liability of the company.
- Shareholders' ownership.
- The independence of the financial disclosure of the company from the financial disclosure of its partners.
- Company manager dismissed.
- Transforming the company into another form.
- Limitations of the powers of the director and partner in the company.
- The responsibility of the company manager for his mistakes towards partners and third parties.
- Exceeding the purposes of the company.
- Neglecting to mention the company.
- Modifications are not documented.
- Violation of the terms of incorporation of the company.
- Non-registration of the company's contract.

The most significant of these is the weakness of guarantees, which is due to the fact that the UAE lawmaker does not set a minimum capital for the company, but rather the legislator is satisfied that the company was established with sufficient capital^25^. As a result of the lack of adequate guarantees in the company, the partners are unable to obtain credit from banks, forcing them to provide a personal guarantee as a guarantee to obtain the credit. Thus their personal liability becomes more than their own money in the event that the company is unable to pay the obligation incurred by it^26^.

**Figure 3:**
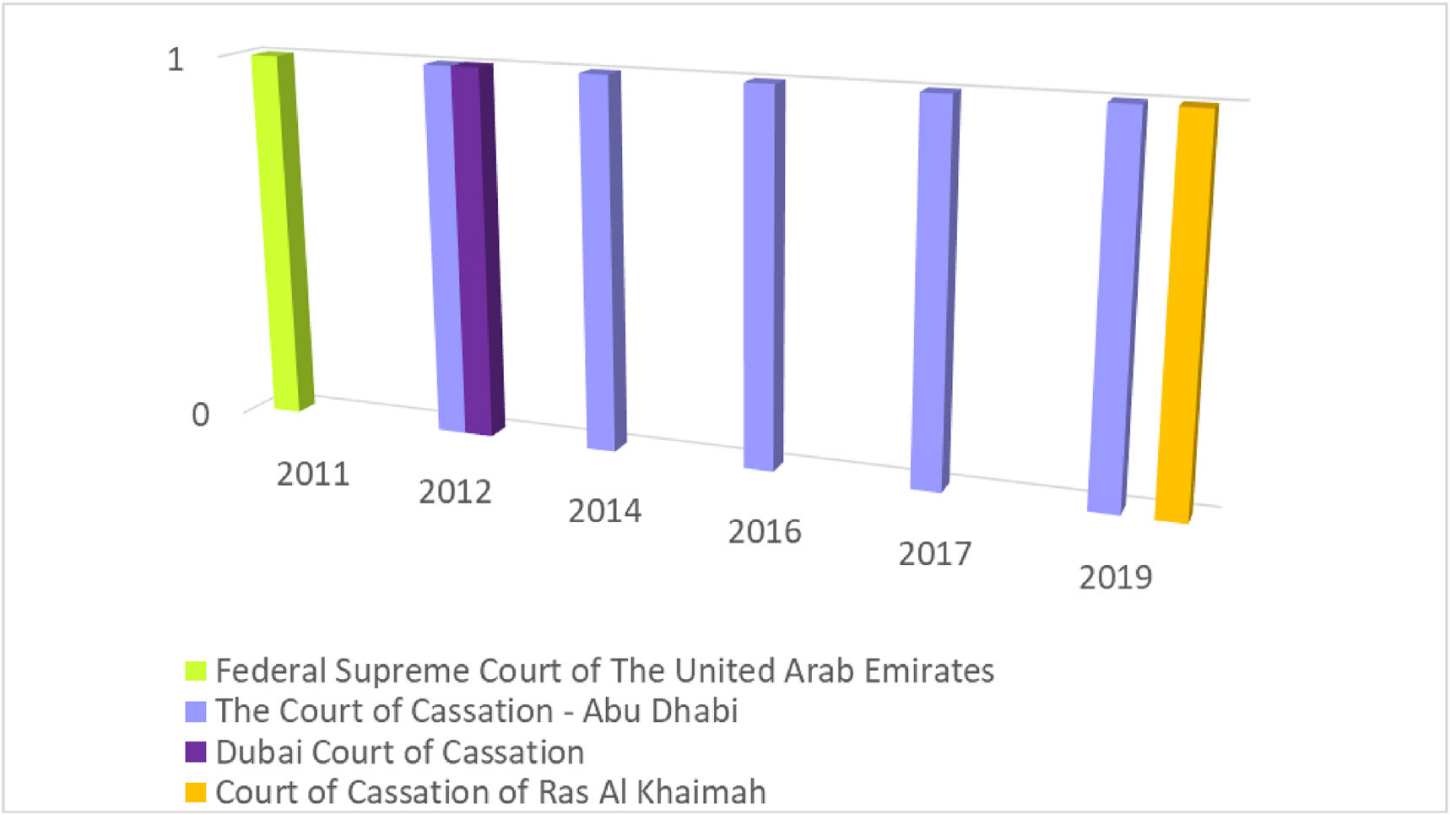
Bankruptcy stats (east law data base).

Figure 3 illustrates the number of Court rulings regarding Bankruptcy at UAE between the years 2010 and 2019 and at every 10 years. It can be noted that Abu Dhabi Court of Cassation was the most court issuing bankruptcy judgments for companies. According to the chart, we find that a bankruptcy judgment was issued in 2012, 2014, 2016, 2017, and 2019 (Figure 3).

As for the Federal Supreme Court of the UAE, there was only one bankruptcy judgment was issued in 2011(Figure 3). In addition, The Dubai Court of Cassation had one bankruptcy judgment was issues in 2014. Similar to the mentioned courts, The Court of Cassation of Ras Al Khaimah had one bankruptcy judgment was issues in 2019 (Figure 3).

**Figure 4:**
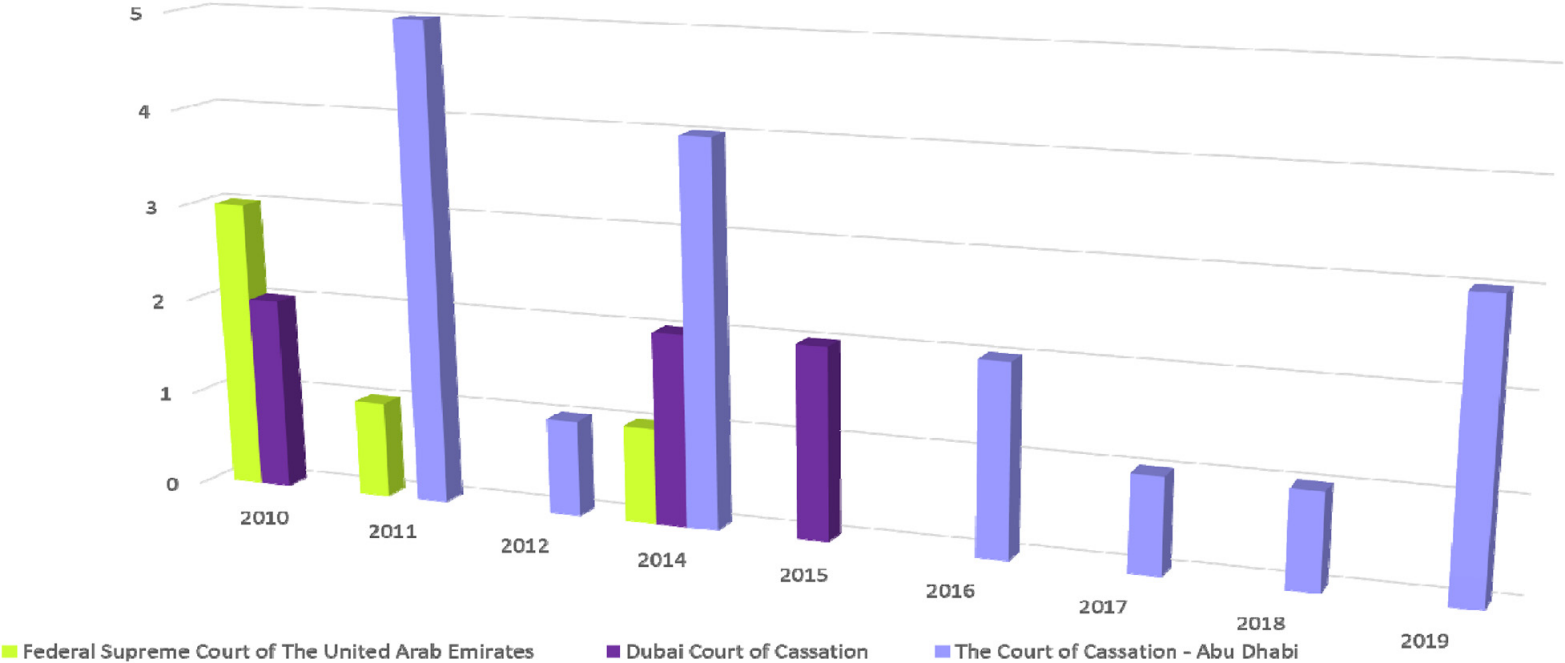
Company litigation stats (east law data base).

Figure 4 illustrates the number of Court rulings regarding Company liquidation at UAE between the years 2010 and 2019 and at every 10 years. It can be noted that most judgments in 2010 were issued by the Federal Supreme Court and the Dubai Court of Cassation. The former issued one judgment on that year, while the latter issued two judgments. It is also noted that the largest number of corporate liquidation judgments was issued by the Abu Dhabi Court of Cassation over the past 10 years (Figure 4). In 2011 there were five liquidation judgments from the Abu Dhabi Court of Cassation, while there was only one judgment from The Federal Supreme Court. In 2012, a single judgment was issued by The Abu Dhabi Court of Cassation (Figure 4).

The Abu Dhabi Court of Cassation swept the largest number of liquidation judgments in 2014, when it issued 4 judgments, while the Dubai Court of Cassation issued two judgments, and the Federal Supreme Court issued a single judgment. In 2015, the only one that issued liquidation judgments was The Dubai Court of Cassation, which issued two judgments that year. From 2016 to 2019, none of these courts issued corporate liquidation judgments, except for The Abu Dhabi Court of Cassation. Where it issued two judgments in 2016, one judgment each in 2017 and 2018, and one judgment in 2019 (Figure 4).

It should be noted that not setting a minimum capital for the company causes people to initiate a limited liability company with a small amount to take advantage of the benefit of limiting liability without taking into account the rights of bona fide third parties dealing with it, thus making it a fertile ground for fraud, deception and splitting of the financial liability to disavow the company's obligations. Despite this, the UAE lawmaker was keen to protect third-party dealers with the company by prohibiting the limited liability company from practicing banking and insurance business or investing money for the account of others^27^. This prohibition has been imposed on this type of companies to prevent them from carrying out speculative activities to protect the rights of creditors and those who invest their money in those operations that involve risks in their practice, while their credit is often weak due to their lack of capital^28^.

## 4 Personal and joint liability of the partner in the limited liability company

The partners believe that they are free from personal and joint liability simply because they are partners in a limited liability company, and therefore they are not liable for its debts and obligations except to the extent of their share in its capital, and the creditors have no guarantee except for the company itself, excluding the personal liabilities of the partners. However, the UAE lawmaker deviated from this principle when it stipulated in the Commercial Companies Law No. 32 of 2021 the cases in which the liability of the partner manager is unlimited on the one hand and the personal and joint liability of the non-manager partner on the other hand.

### 4.1 Not indicating the type of company when contracting with others

The Emirati lawmaker obligated the director or directors of the company to include the phrase “with limited liability” and “indicating the amount of the company's capital”^29^ in the company’s official documents and publications to protect those dealing with the company. The wisdom behind the requirement to mention the form of the limited liability company is the legislator’s keenness to inform others of the nature of the company in which they deal with and to protect it from the misconception that it is a company of persons in which the partners whose names are mentioned in the address of the company are personally and jointly responsible for the debts and commitments of the company^30^. The director or directors of the limited liability company are responsible for monitoring the name of the company and ensuring that it conforms to the provisions of the law, and in the event of a breach of this obligation, they shall be responsible for the company's obligations with their own funds and jointly, in addition to compensation if required^31^.

In applying this, the Court of Cassation in Abu Dhabi ruled that “without mentioning the statement that the company is a “limited liability company” and the amount of its capital, the manager should be responsible with his own funds for all obligations arising from the action concluded with the company as if he were a joint partner.” This liability is limited to those who intervene in this behavior, and it is not realized unless it is proven that this omission caused harm to those who dealt with this company, provided that the third party who dealt with it proves that harm was caused as a result of this negligence that was the productive cause effective to cause the harm that should be contemporized to dealings occurring between the two parties”^32^.

However, the liability of the managing partner or the partners as a whole in this case is contingent on the fact that others do not know the truth about the company and that it has limited liability, as well as the injury of others as a result of their lack of knowledge about the company’s reality during their dealings with it. If a third party is able to learn the truth about the company from its partners, or if they deal with it on this basis, or if it is not harmed as a result of this dealing, then this penalty is not applicable^33^.

As for the one-person company, Article 72 of the Commercial Companies Law stipulates that the name of the company must be associated with the name of its owner and followed by the phrase “a single person company with limited liability”, and in application of this, the sole partner will be personally liable if he chooses a name for the company derived from its purpose and does not associate it with his personal name, or associates its name to the name of a third party, or its name was not followed by the term “one-person limited liability company^”^^34^.

**Figure 5:**
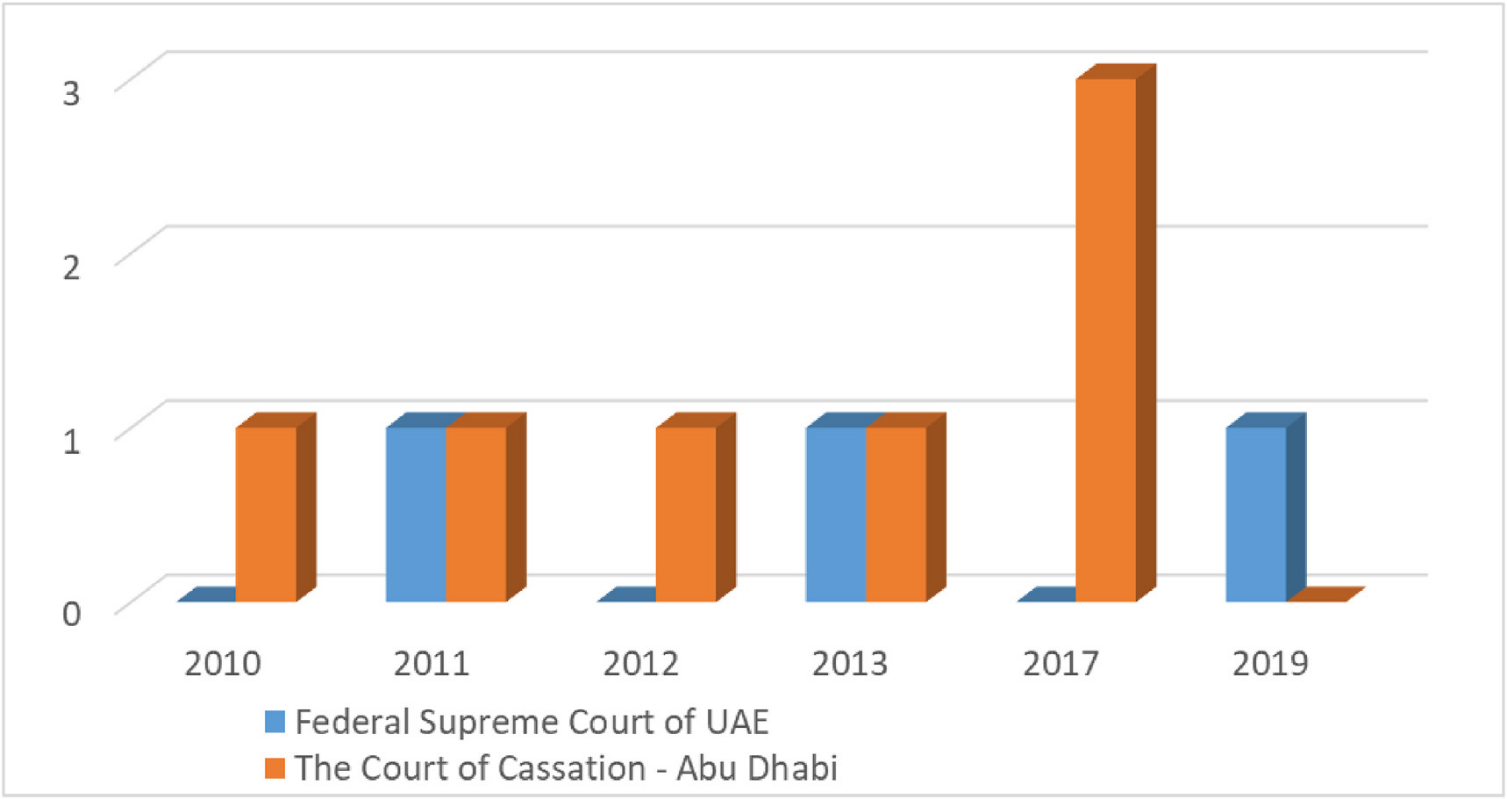
Not indicating the type of company (east law data base).

Figure 5 illustrates the provisions issued against companies due to neglecting to mention the company's types at United Arab Emirates between the years 2010 and 2019 and at every 10-years.

It can be noted that Abu Dhabi Court of Cassation was the most court issuing provisions against companies due to neglecting to mention the company's description. According to the chart, we find that there is a single judgment issued in 2010, 2011, 2012, and 2013 (Figure 5). Nevertheless, as we can notice at 2017, there was 3 judgments regarding the same issue. As for The Federal Supreme Court of the United Arab Emirates, there was only a single judgment was issued in 2011, 2013, and 2019 (Figure 5).

### 4.2 Not registering the company's articles of incorporation and any amendment to it

The registration of a limited liability company requires certain formal procedures. It is clear that the UAE lawmaker has referred to these procedures for the registration of a joint liability company^35^. Article 43 (1) stipulates that the competent authority shall specify the data and documents necessary for the establishment of the company. The competent authority shall then develop, in accordance with the provisions of the law, a form for the incorporation application, and the partners shall submit the application to establish the company to the competent authority, accompanied by the documents required for licensing and registration. The competent authority may request the partners to complete whatever data and documents it deems necessary to submit, or make amendments to the company's articles of incorporation. In the event that all the required documents are completed, the competent authority shall issue its decision on the company’s registration application^36^.

If the competent authority approves the incorporation of the company, the company contract and every amendment that occur to it must be registered with the competent authority until the contract is deemed effective vis-a-vis others. Failure to register the contract results in non-enforcement of it vis-a-vis others.

**Figure 6:**
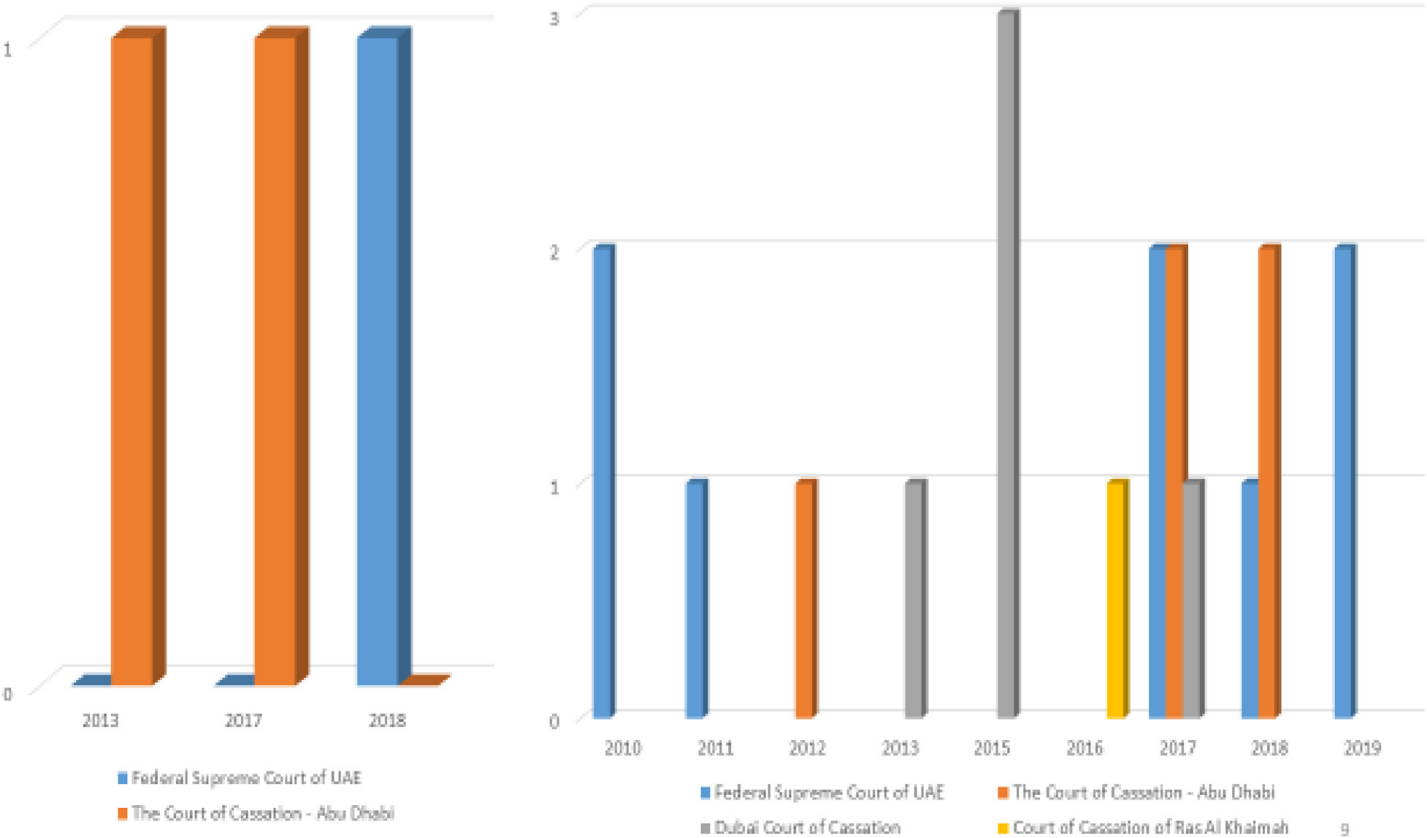
Not Registering the Company's Articles of Incorporation and any Amendment to it (East Law Data Base).

Figure 6 illustrates the provisions issued against companies due to Factual Companies and the amendments that are not officially documented. At UAE between the years 2010 and 2019 and at every 10 years. For the first chart regarding “Modifications are not documented”, It can be noted that Abu Dhabi Court of Cassation was had issued a judgment in 2017, and 2018. As for The Federal Supreme Court of the UAE, there was only a single judgment issued in 2019 (Figure 6). For the Second chart regarding “Factual Company”, It can be noted that Dubai Court of Cassation had issued the most judgments at 2015, as it issued 3 judgments at the same year. While in 2013 and 2017, it had issued a single judgment each (Figure 6). As for the Federal Supreme Court of UAE it issued two judgments in 2010, 2017, and 2019. It shows that only one judgment each in 2011 and 2018 (Figure 6).

For the Court of Cassation – Abu Dhabi it issued two judgments in 2017 and 2018, and only a single judgment in 2012. While the Court of Cassation – Ras Al Khaimah issued a single judgment in 2016 (Figure 6).

It should be noted that violating the registration procedures during the incorporation stage does not forfeit the right of the one who was harmed by resorting to the judiciary to claim compensation. In the event that the court decides to invalidate the company, any affected person shall have the right to demand compensation from the founders for the harm caused, and this does not mean depriving the injured from not observing the laws governing companies of their right to file a lawsuit, as long as the violation has caused harm to the partners or other company’s stakeholders, even if a judgment of nullity has not been issued^37^. Article 15 (4) also clarifies the joint liability of the director or members of its board of directors of the company to compensate for the damage suffered by the company, partners or others due to the failure to register the company and any amendments to it in the commercial register with the competent authority.

**Figure 7:**
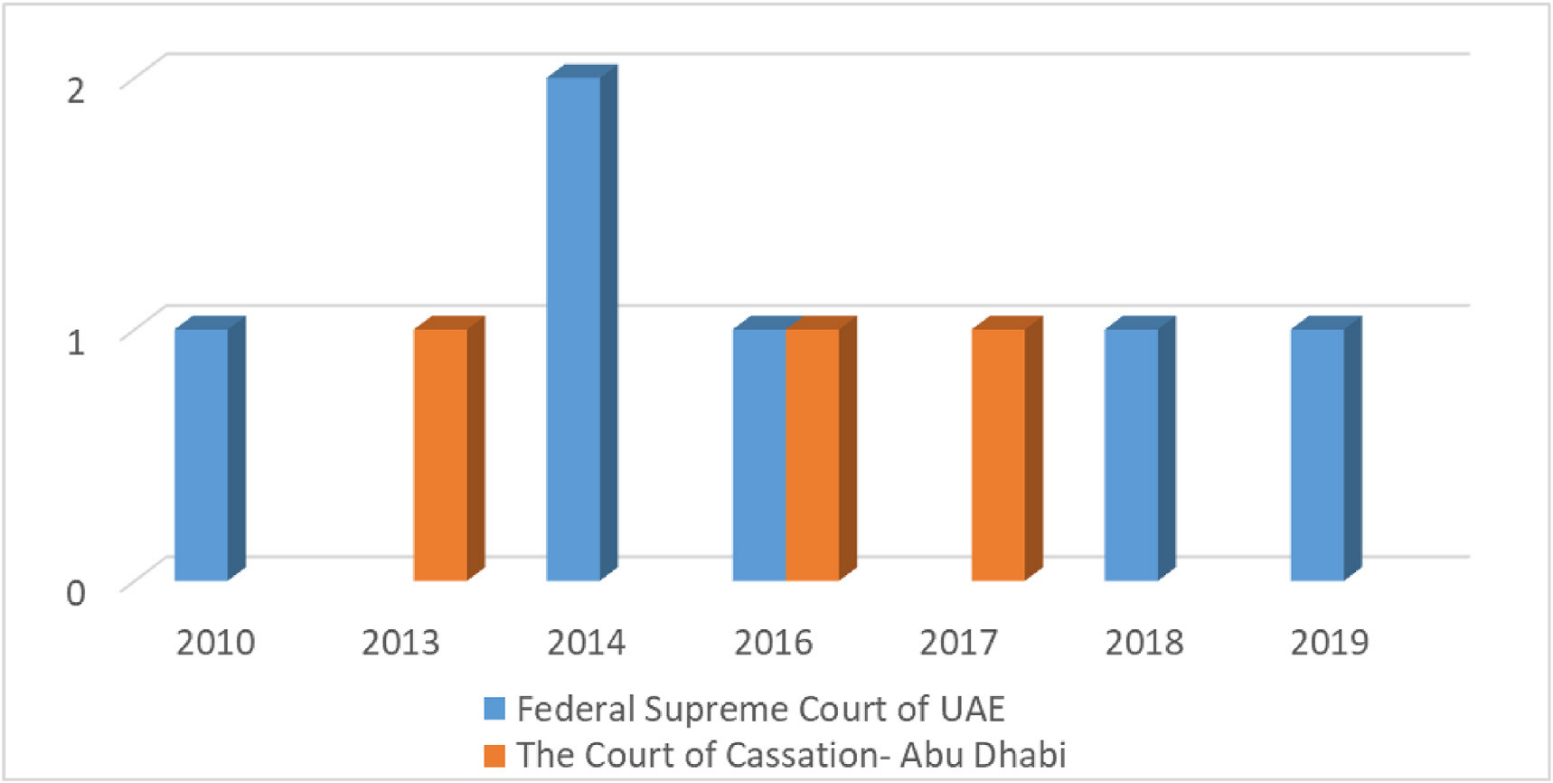
Violation of the terms of incorporation of companies that incurs harms to third party (east law data base).

Figure 7 illustrates the provisions issued against companies due to violation of the terms of incorporation of companies that incurs harms to third party. It can be noted that The Federal Supreme Court of UAE had issued 2 judgments in 2014, and a single judgment issued in 2010, 2016, 2012, 2018, and 2019 (Figure 7). While The Court of Cassation - Abu Dhabi had issued a single judgment in 2013, 2016, and 2017 (Figure 7).

### 4.3 Not establishing the company in accordance with the law

Article 9 (1) specifies the forms that a commercial company must take, which are: a joint liability company, a limited partnership company, a limited liability company, a public shareholding company, and a private joint stock company. Unless the legislature restricts the practice of some commercial activity in a specific form of company, the partners have the freedom to choose the form of the company that best meets their interests and allows them to practice the activity they wish to practice. But if the company was not properly incorporated and properly registered in the commercial registry, and did not acquire the legal personality as a limited liability company, then its legal system, especially the liability system in it, does not apply to the partners, but in this case it is considered an actual company and is subject in this case to the ruling of Article 9 (2) whereby all persons who contract in its name are considered personally and jointly responsible for the obligations arising from this contract^38^. In implementation of this, the Federal Supreme Court came to the conclusion that “it is decided in the judiciary of this court that the limited liability company has a moral personality and a financial liability that is independent of the persons and liabilities of its partners, and that it is liable on its own for all debts that arise as a result of its dealings with others in its commercial activity and that the partner who pays his share in it is safe from third parties claiming the debts of the company. The exception to this rule is a breach of the company’s incorporation rules, its declaration and its documentation with the official authorities, at which time all partners are considered bound by mutual obligation in their own funds for the company's obligations^39^.

If the company’s articles of incorporation include any wrong or incorrect data regarding the partners ’contribution to the company's capital, whether in cash or in kind, this is due to the fact that Article 8 of the Companies Law indicated that two or more persons provided a share of money, as well as Article 76 of the Companies Law requires that the partners contribute to the company with its capital, whether in cash or in kind, and that contribution must be paid in full at the time of its incorporation because this represents the company's capital and it is the only guarantee for the company's creditors. Therefore, the shareholders’ liability is limited to that. False or incorrect data in the articles of incorporation of the company, is considered a violation of the principles of the company’s incorporation and may lead to its nullity, which in some cases leads to the company being considered an actual company in which the partners are personally and jointly responsible for all its obligations and debts.

**Figure 8:**
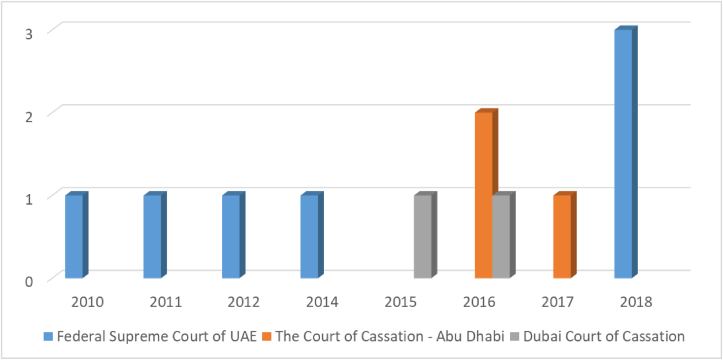
Not establishing the company in accordance with the law (east law data base).

Figure 8 Illustrates the provisions issued against companies due to violation of the terms of incorporation of companies.

It can be noted that The Federal Supreme Court of UAE had issued 3 judgments in 2018, and a single judgment issued in 2010, 2011, 2012, and 2014 (Figure 8). While The Court of Cassation - Abu Dhabi had issued 2 judgments in 2016, and a single judgment in 2015 (Figure 8). As for Dubai Court of Cassation had issued a single judgment in each of 2015 and 2016 (Figure 8).

### 4.4 Third party proof of the company' articles of incorporation

The lawmaker stipulated that the company contract should be in writing, otherwise the contract is void, even if this nullity is of a special kind^40^, because the court cannot rule it on its own, but rather it must be requested judicially, it is permissible for everyone who has an interest to cling to the nullity for not writing^41^. In this context, Article 14 of the UAE Commercial Companies Law states that “the company incorporation contract and every amendment that occurs to it must be drawn up in the Arabic language and notarized before a notary public, otherwise the contract or amendment is void”. The position of the Emirati lawmaker on writing is that it is a pillar of the meeting and not a requirement for proof only, as there is no company without it^42^.

Failure to write the company’s contract results in the nullity of the contract, and therefore it is permissible for others to prove the company’s contract or amend it by all means of proof, and others have the right to cling to the existence of the company or its nullity vis-à-vis the partners, and whoever has contracted with others in the name of the company that is judged to be null at the request of others shall be liable for personal and joint liability for its obligations arising out of this contract, provided that the debtors of the company have no right to claim the company’s nullity or to adhere to it to get rid of the debts owed by them^43^. The Federal Supreme Court affirmed that “adherence to the existing contract of the company between the partners at the time of the transaction, even if this contract has not been publicized or registered in the commercial register does not affect the failure to prove the company’s establishment in the facility’s license, and that the formal or null partnership contract is not invoked except among the partners, and for others to adhere to the apparent contract of the company. If the commercial company starts its activities but has not been registered, and the month of its contract is in accordance with the law, it is nevertheless considered existing in relation to others as a de facto company and has - as such - a legal personality and a financial liability of its own and independent of personality and debts of partners in it, and these will be jointly responsible among themselves - and with it - for the actions of one of the partners - as an agent for the rest of the partners in carrying out the company's business”^44^.

### 4.5 Exceeding the upper limit for the number of partners

The UAE lawmaker has limited the number of partners in limited liability companies to fifty^45^. Thus, the lawmaker has been keen to preserve the personal character of the company and distinguish it from the joint-stock company, which does not have a ceiling because it is one of the associations of capital that does not care about the personality of the partner as much as it does about its money. As confirmation from the lawmaker to retain the personal character of the company, Article 75 (1) stipulates that if the number of partners exceeds fifty, the manager or managers must, according to the circumstances, notify the competent authority within (30) thirty days from the date of that increase, and the company must correct its status within three months from the date of notification, otherwise the company is considered dissolved, and the partners in it will be personally liable for their money and jointly between them for the debts and obligations owed by the company from the date of increasing the number of partners.

It is noted from the text of Article 75 (2) of the Companies Law that the UAE lawmaker stipulated a clear and explicit provision in the event of increasing the number of partners, which is the joint liability of the partners, but it is an exceptional situation in which the partner is not responsible, the first of which is that the increase in the number is a result of the transfer of ownership of shares to more than one person, through inheritance or a final court ruling, the second of which relates to the partners who are proven to have no knowledge of, or objection to, the increase.

Article 75 of the Companies Law also deals with the case in the number of partners exceeding the maximum in two ways: The first is to correct the company's conditions within the legally mandated time frame, which results in the company continuing to operate. The second is the company's termination by force of law in the event that the company is unable to correct its situation. An opinion – which we agree with - goes on the existence of a conflict between what is stated in Article 75 of the Companies Law regarding the termination of the company in the event that the conditions of the company are not corrected and Article 275 of the Companies Law that allows the company to convert to another form, i.e. the survival of the company, but in another form other than a company with limited liability^46^.

**Figure 9:**
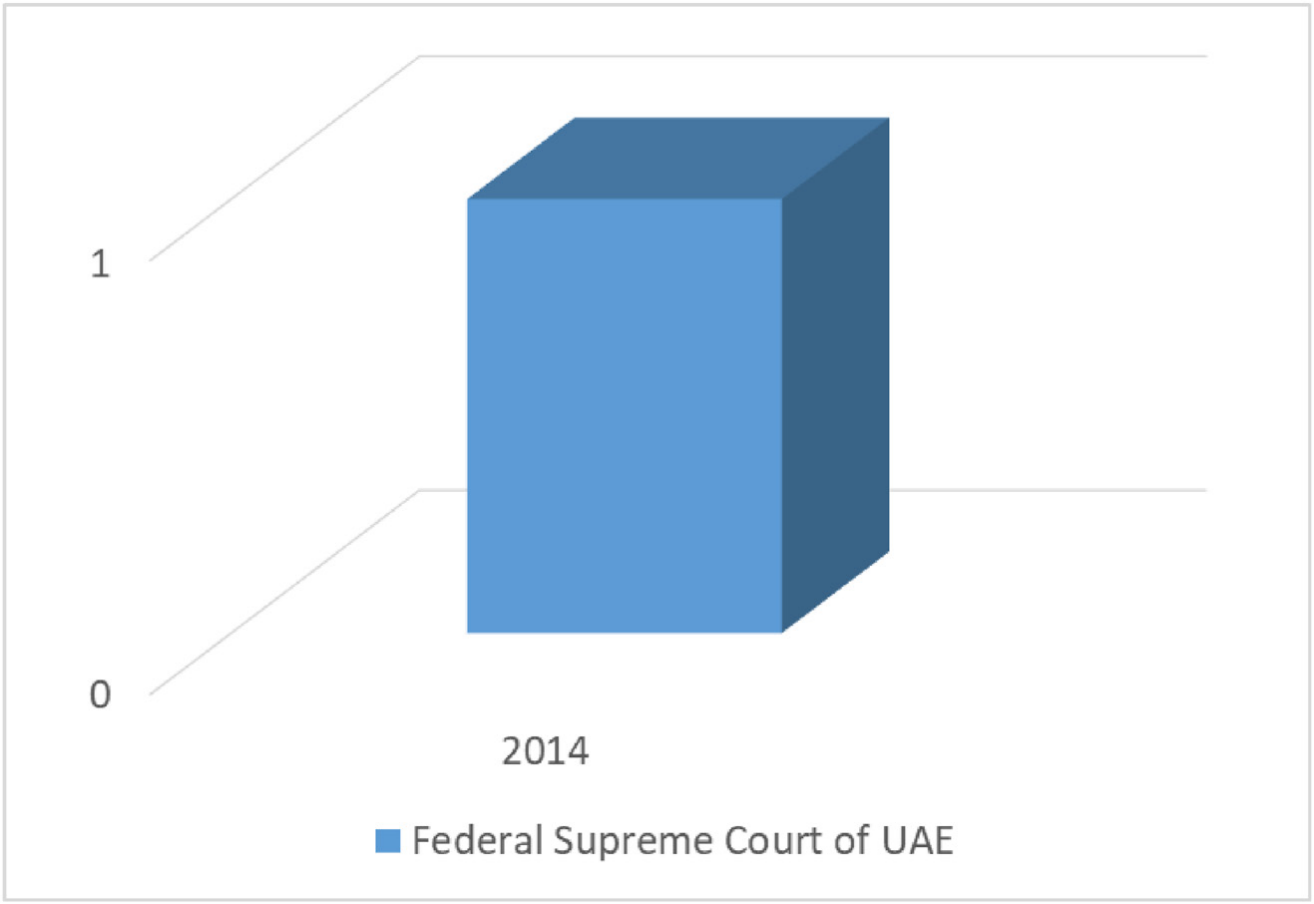
Exceeding the upper limit for the number of partners (east law data base).

Figure 9 illustrates the provisions issued against companies due to exceeding the maximum number of partners. For the chart regarding Violation of the maximum number of partners, The Federal Supreme Court of the United Arab Emirates issued a single judgment in 2014 (Figure 9).

### 4.6 Wrong evaluation of the in-kind share at a value higher than the real value

The capital of a limited liability company consists of cash and in-kind shares. It is not permissible to present work as a share in the company's capital based on the text of Article 17 (2) of the Companies Law, which limits the provision of the share to work on the joint partner.

In Article 78 (1) the UAE lawmaker permitted the partners in the limited liability company to provide an in-kind share in the company.

To ensure that the partner does not overestimate the in-kind share by the Emirati legislator, Article 78 (2) of the Companies Law stipulates the method of evaluating the in-kind share in the limited liability company through one or more financial advisors approved by the Securities and Commodities Authority who are selected by the Authority at the expense of the Company. While Article 78 (4) referred to an exceptional case, where the lawmaker permitted the evaluation to be carried out by the partners, in which case the competent authority must approve this value. The reason for allowing partners to assess the in-kind share is to avoid the partners' administrative procedures that impede the establishment of the company, as well as exempt the partners from financial expenses that may exceed the value of the in-kind share itself^47^.

In Article 78 (4) the lawmaker considered that the provider of the sample share is responsible for the correct assessment of the value of the in-kind share, and arranged a penalty for violating this, which is the commitment of the partner providing the share to pay the difference in cash to the company in the event that it is proven that the in-kind shares were estimated at more than their real value.

It should be noted that the old companies' law^48^ used to assess the liability of the share provider with the founders of the company for the difference between the two values, but the current companies' law made the liability to pay the difference on the share provider. Therefore, it made the partners' approval of the estimate formal, as it established the full liability on the partner providing the share.

The question arises about the type of responsibility imposed by the lawmaker on the partner providing the share, where an opinion went to consider that the exaggeration in the estimation of the in-kind share is not a tort or contractual liability, but rather it is a liability based on a breach of a legal obligation to guarantee that the law imposes on the partners to ensure the safety of the capital to the creditors of the company^49^. We see that what is meant by the liability contained in Article 78 of the Commercial Companies Law is the personal and joint liability in paying the difference in cash, as this guarantees the rights of others on the part of the company. In addition, the lawmaker imposed criminal responsibility in assessing the in-kind share in excess of its value. Article 347 of the Commercial Companies Law provided that “shall be punished by imprisonment for a period of not less than six months and not exceeding three years and by a fine of not less than five hundred thousand dirhams and not more than one million dirhams or one of these two penalties whoever ill-intentionally values the in-kind shares provided by the founders or partners in excess of their real value.”

**Figure 10:**
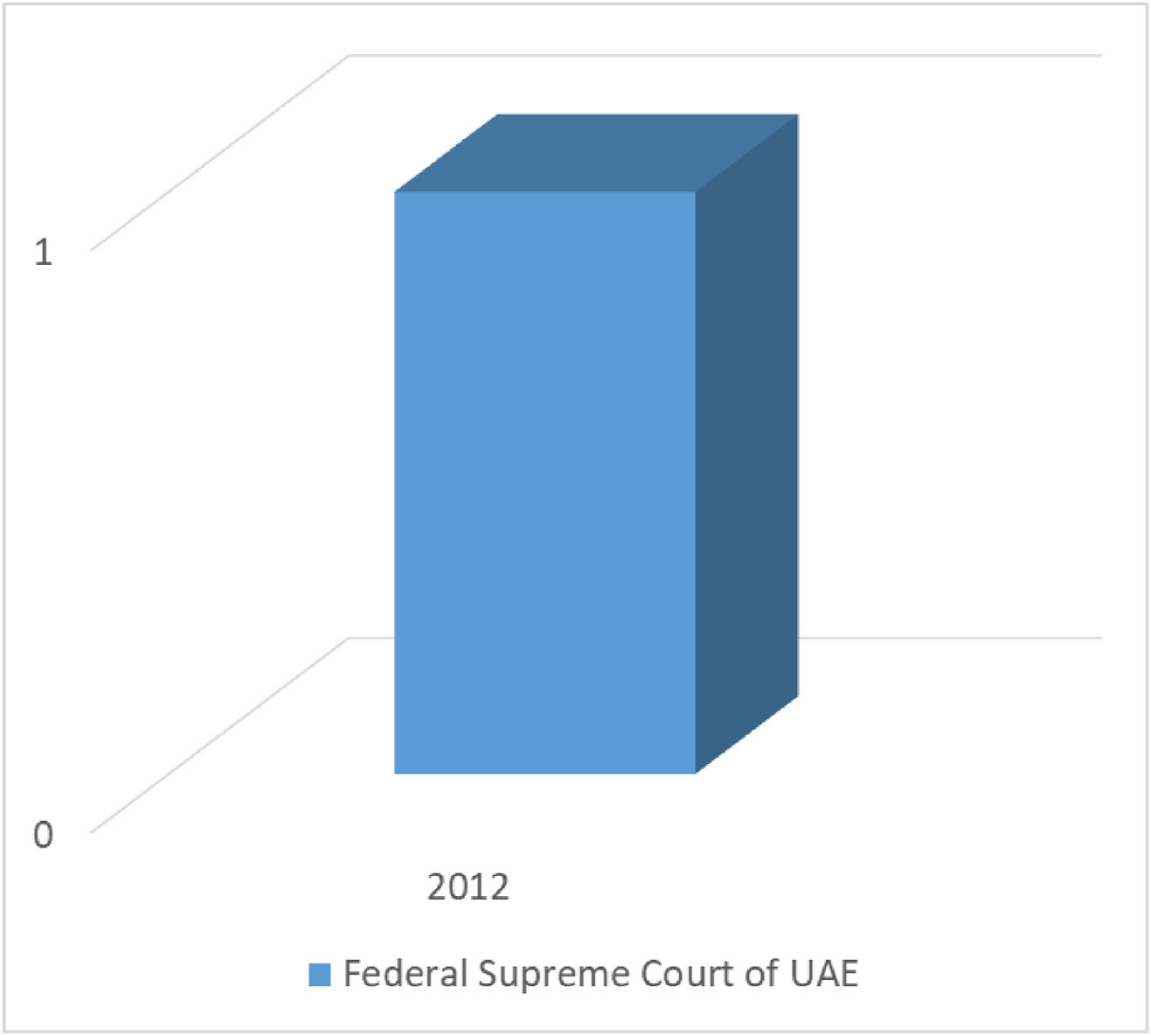
Evaluation of the in-kind share at a value higher than the real value (east law data base).

Figure 10 illustrates the provisions issued against companies due Re-evaluation of the in-kind stake in the company. For the Chart about Re-evaluation of the in-kind share in the company, issued a single judgment in 2012 (Figure 10).

### 4.7 The loss reaches half of the company's capital

In Article 308 of the Companies Law, the Emirati lawmaker required managers in a limited liability company, in the event that the loss reaches half of the capital, to present the matter to the general assembly to consider the dissolution of the company based on a decision by the majority. If the loss reaches three quarters of the capital, the lawmaker has granted the owners of a quarter of the capital to dissolve the company^50^.

If the managers decide to support and continue with the partners without referring to the general assembly, then the liability of the partners is personal and joint, as the directors of the limited liability company are subject to the provisions related to the members of the board of directors of the public joint-stock company^51^. With reference to the provisions of the public shareholding company, we find that the lawmaker stipulated in Article 162 of the Companies Law that “members of the board of directors are responsible towards the company, shareholders and others for all acts of fraud and abuse of power, and for every violation of the law and the company's system, and for error in management, and every condition that provides otherwise is null. He shall rule otherwise”. It is understood from the previous text that the liability of the managers in the limited liability company is personal and joint towards the company, the partners and others, but if the mistake is made by consensus of the partners in the limited liability company, then the liability of the company is jointly between them for this error. But if the decision was issued by the majority, the opponents will not be asked about it when they have proven their objection to the decision during the session and it was recorded in the session minutes. If one of the partners is not present at the decision-making session, his liability remains unless it is proven that he was unaware of the decision or was aware of it despite his inability to object to it^52^.

## 5 Conclusion

In this paper, we reviewed the liability of the partners in the limited liability company according to the UAE Commercial Companies Law No. 32 of 2021. The study concluded that the limited liability company is one of the companies most commonly used and preferred by all investors in the United Arab Emirates (in terms of legal form), whether they are local or foreigners, for several reasons, the most important of which is that it has a legal personality independent of the partners in the company and whose liability is limited to the extent of their contribution to its capital. Article 21 of the Companies Law states that the company acquires independent legal personality as of the date of its registration in the commercial register with the competent authority, and Article 71 of the Companies Law states that each partner in the company is responsible to the extent of his share in the company's capital.

Nevertheless, the study concluded that the liability of the partner or partners in this company may shift from being limited by the amount of their contribution to its capital to a personal and joint liability where they may ask about their own money towards the company's obligations, where all the partners in it are responsible for all debts and obligations of the company.

In addition, the study concluded that if the company was not properly incorporated according to the law or a ruling of its nullity, the partners who contracted with third parties on behalf of the company will be personally and jointly responsible for all its obligations arising from those contracts, if the company’s articles of incorporation include any wrong or incorrect data, with regard to the share of partners in the company’s capital. The lawmaker also dealt with a case where the number of partners in the company after its incorporation exceeded the limit stipulated in Article 71 of the law (which is 50 partners) and the company did not correct that situation by reducing the number of partners in it to the maximum stipulated in by law, within a maximum period of 3 months after receiving a notification from the local authority, the company is considered dissolved and the partners will be personally and collectively responsible for its emerging debts and obligations.

Indeed, the study concluded that if the contribution to the company's capital by one of the partners is an in-kind share and the valuation of that in-kind share exceeds its real value, then the partner who provided that share must pay the difference in cash to the company, and this partner will be personally responsible towards others for any difference that appears between the valuation and the real value of that share.

## Declarations

### Author contribution statement

Dr. Nazzal Kisswani: Conceived and designed the experiments; Performed the experiments; Analyzed and interpreted the data; Contributed reagents, materials, analysis tools or data; Wrote the paper.

Dr. Ahmad Farah: Analyzed and interpreted the data.

### Funding statement

This research did not receive any specific grant from funding agencies in the public, commercial, or not-for-profit sectors.

### Data availability statement

Data included in article/supp. material/referenced in article.

### Declaration of interests statement

The authors declare no conflict of interest.

### Additional information

No additional information is available for this paper.

## Footnotes

1 It combines the characteristics of the capital associations in terms of their management and determining the liability of the partners in them, and the characteristics of the people’s companies in terms of the number of partners and the mutual trust between them. According to the UAE Companies Law No. 32 of 2021, it includes the limited liability company.

2 It is mainly based and primarily on the personal consideration, as it usually arises between a small number of people who are united by the bond of kinship or friendship and each puts his trust in the other so that the personality of each partner is the subject of consideration of the other partners, so that the company will terminate as a general rule in the event that this consideration is removed. According to the UAE Commercial Companies Law No. 32 of 2021, it includes both a joint liability company and a partnership company. See also: Farah, A. Q. (2015). Réflexions critiques sur le statut juridique de la société en nom collectif en droit émirati à la lumière du droit français, Arab Law Quarterly, 29(2), 138–167.

3 This company is based on financial consideration and not the personality of its constituent partners. According to the UAE Commercial Companies Law No. 32 of 2021, it includes both a public shareholding company and a private shareholding company.

4 The present Decree-Law was published in the Official Gazette of the United Arab Emirates, issue no. 712 Annex.

5 Sherif M. Ghanem and Saleh R. Al-Hamrani, Explanation of the New Commercial Companies Law No. 2 of 2015, Police Academy, 2017, p. 445.

6 Fawzi M. Sami, Commercial Companies: Public and Private Provisions, House of Culture, Jordan, 2012, p. 180. The English law went to designate it as a private company defined by shares in order to distinguish it from the joint-stock company that puts its shares for subscription, and the Jordanian Companies Law of 1964 named this type of the companies the private shareholding company, and in the repealed law of 1989 and the law of 1997 took the French name and called it the limited liability company.

7 Article 364 of the Commercial Companies Law No. 32 of 2021 stipulates that “The aforementioned Federal Law no. (2) of 2015 shall be abrogated, as well as any provision that contravenes or contradicts the provisions of this Decree-Law.”

8 Article 3 of the UAE Commercial Companies Law No. 32 of 2021 stipulates that “The provisions of this Decree-Law, as well as the regulations and decisions issued in implementation thereof, shall apply to commercial companies established in the State; also, the provisions relating to foreign companies set forth in this Decree-Law, as well as the decisions and regulations issued in implementation thereof, shall apply to foreign companies that establish their head office in the State for the practice of any activity therein or establish a branch or representative office thereof in the State.”.

9 Article 12 of the UAE Commercial Companies Law No. 32 of 2021.

10 Article 9 of the UAE Companies Law stipulates that “1. The company must take one of the following forms: A- Joint liability, B- Limited Partnership, C- Limited Liability, D- Public shareholding, E- Private shareholding.

11 Article 13, Paragraph 2 of the UAE Commercial Companies Law No. 32 of 2021.

12 Article 71 of the UAE Commercial Companies Law No. 32 of 2021 stipulates that “1- A Limited Liability Company is a company where the number of partners is at least two (2) but shall not exceed fifty (50). A partner shall be liable only to the extent of its share in the capital. 2- A single natural or corporate person may incorporate and hold a Limited Liability Company. The holder of the capital of the company shall not be liable for the obligations of the company other than to the extent of the capital as set out in its Memorandum of Association. The provisions of the Limited Liability Company contained in this Law shall apply to such person to the extent not in conflict with the nature of the company”.

13 Suzan A. Hussain, The Limited Company - Really a bulwark from the unlimited liability of the partners: An Analytical Study of the Texts of the Commercial Companies Law No. 2 of 2015, Journal of Security and Law - Dubai Police Academy, 2016, p. 317.

16 Samiha Al-Qalioubi, Commercial Companies, Dar Al-Nahda Al-Arabiya - Cairo, 2014, p. 487.

17 Federal Supreme Court, Civil and Commercial Judgments, Appeal No. 49 of 2012 Judicial dated 04-23-2012.

18 Samiha Al-Qalioubi, previous reference, p. 488.

19 Suzan A. Hussain, previous reference, p. 328.

20 Ala’a Y. Yusef, Rules of a One-Person Company between Determining the Liability of the Partner or Not: A Legal Study, University of Sharjah, Journal of Legal Sciences, Vol. 17, Issue 2, 2020, pg. 206.

21 Rasha M. Hattab and Ahmed Q. Farah, Commercial Companies in the United Arab Emirates, University of Sharjah, 2017), p. 74.

22 Alaa Y. Yusef, previous reference, p. 208.

23 Federal Supreme Court, Civil and Commercial Judgments, Appeals No. 513 and No. 579 of Judicial Year 25 dated 6-27-2004.

24 Sharif M. Ghanem and Saleh R.Al-Hamrani, previous reference, p. 460.

25 Article 76, Paragraph 1 of the UAE Commercial Companies Law No. 32 of 2021 stipulates that “The company shall have sufficient capital to achieve the purpose of its incorporation and the capital shall consist of shares equal in value. On a proposal made by the Minister in coordination with the competent authorities, the Cabinet may issue a decision determining the minimum limit of the capital of the company.”

26 Suzan A. Hussain, previous reference, p. 338; see also Alaa Y. Yusef, previous reference, p. 225.

27 Article 11, paragraph 3, of the Commercial Companies Law No. 32 of 2021 stipulates that “4- Only Public Joint Stock Companies may conduct banking and insurance activities. Only Joint Stock Companies may invest money for the account of third parties.”

28 Ruling of the Kuwaiti Court of Cassation - Commercial Division - No. 116 of 1987, on the hearing of 1-4-1988.

29 Article 12 of the UAE Commercial Companies Law No. 32 of 2021 stipulates that “2- All contracts, quittances, correspondence and application forms issued by the company must bear its name, legal form, registration number and address, and if the amount of its capital is added to these data, the amount of the paid capital must be indicated.”

30 Rasha M. Hattab and Ahmad Q. Farah, previous reference, p. 346.

31 Article 72 of the UAE Commercial Companies Law No. 32 of 2021 stipulates that “1- A limited liability company shall have a name derived from its purpose, or from the name of one or more of the partners, provided that the name ends with the phrase” a limited liability company “and an abbreviation with the phrase (LLC). In the case of a one-person company, the name of the company must be associated with the name of its owner and followed by the phrase a single person company with limited liability.2- If the manager - or the managers - violated the provisions of Clause (1) of this Article, they were responsible for their own money and jointly for the obligations of the company, in addition to the compensation if required.

32 Abu Dhabi Court of Cassation - Civil and Commercial Judgments - Appeal No. 725 of 2016 Judicial - Commercial Department – 1, 31, 2017 Technical Office 11 Part No. 1 Page No. 348.

33 Sharif M. Ghanem and Saleh Rashid Al-Hamrani, previous reference, p. 467.

34 Alaa Y. Yusef, previous reference, p. 215.

35 Article 73 (1) of the UAE Commercial Companies Law No. 32 of 2021 stipulates that “a limited liability company shall be established and registered as stipulated in Articles (42 and 43) of this law.”

36 Article 43 of the UAE Commercial Companies Law No, 32 of 2021 stipulates that “2. The incorporation application shall be submitted to the competent authority together with the documents necessary for licensing and registration. 3. The competent authority shall instruct the applicant to complete whatever data and documents it deems necessary to submit or make amendments to the company contract in a manner that makes it consistent and the provisions of this law and the decisions issued pursuant thereto 4. The competent authority shall issue its decision regarding the application for establishing the company within a maximum period of (5) five working days from the date of submitting the application or completing the data and documents or making the amendments requested by it, and in case the request is rejected, the refusal must be justified.

37 Akram Yamlaki, Commercial Law (Corporate), a Comparative Study, House of Culture for Publishing and Distribution - Amman, 2006, p. 191.

38 Article 9, paragraph 2, stipulates that “every company that does not take any of the forms referred to in the previous clause shall be deemed null and the persons who contracted in its name shall be personally and jointly responsible for the obligations arising from this contract.” See also Sharif Ghanem and Saleh Al-Hamrani, previous reference, p. 467.

39 Federal Supreme Court - Civil and Commercial Judgments - Appeal No. 265 of 2011, Judicial dated 22-11-2011.

40 Basem Melhem and Bassam Al-Tarawneh, Explanation of the New Qatari Commercial Companies Law, First Edition - without publisher, 2016, p. 52.

41 Rasha M. Hattab and Ahmed F. Qassem, previous reference, p. 59.

42 Salama F. Arab, the Mediator in Commercial Law: Part Two, Commercial Companies, Cairo, Dar Al-Nahda Al-Arabiya, 2009, p. 105.

43 Article 16 of the UAE Companies Law No. 32 of 2021 stipulates that “1. A third party may prove the contract of the company or any amendment that occurs to it by all means of proof and it may also insist on the existence of the company or its invalidity vis-à-vis the partners. 2. If the company is judged to be null at the request of others, it is considered as if it was never existed for him, and the persons who contracted with him in the name of the company are jointly and personally liable for the obligations arising from this contract. In all cases where the company is judged null and void, the company’s liquidation and the settlement of partners ’rights shall apply to some of them before some of the terms of the contract. It is not permissible for the debtors of the company to seek nullity or insist on it in order to get rid of their debts before the company.

44 Federal Supreme Court, Civil and Commercial Judgments, Appeal No 79 of Judicial Year 21 dated 20/12/2000.

45 Article 71, paragraph 1, stipulates that “a limited liability company is a company in which the number of partners is not less than two and no more than (50) fifty partners, and each of them is only liable to the extent of his share in the capital.”

46 Suzan A. Hussain, previous reference, p. 367.

47 Mustafa K. Taha, Commercial Companies, University Press, Alexandria, 2000, p. 423, see also Rasha Hattab and Ahmed Qassem, previous reference, p. 353.

48 Article 223 of the repealed UAE Commercial Companies Law of 1984 stipulates that “if a partner submits an in-kind share, its value must be assessed in the company’s articles of incorporation with an indication of its type, the name of its provider, and the amount of capital it represents. The provider of the share shall be responsible before others for the correct estimation of its value in the company's contract. If it is proved that the share was estimated at more than its real value, the share provider must pay the difference in cash to the company, and the founders will be responsible for their jointly owned funds for paying this difference.

49 Mustafa K. Taha, previous reference, 423, see also Rasha Hattab and Ahmed Qassem, previous reference, p. 353.

50 Article 308, Paragraph 2 of the Commercial Companies Law stipulates that “if the loss reaches three quarters of the capital, partners in possession of a quarter of the capital may request dissolution.

51 Article 84 of the UAE Companies Law No. 32 of 2021.

52 Article 162, Paragraph 2 of the UAE Commercial Companies Law No. 32 of 2021.

## Footnotes

14 Article 32 of the UAE Commercial Companies Law No. 32 of 2021 stipulates that “No company other than a public Joint Stock Company may offer any Securities for public subscription. In all events, no company, entity, natural or corporate person incorporated or registered inside the State or in the free zones or abroad may publish any advertisements in the State, including the invitation for public subscription in Securities without the prior consent of the authority”.

15 Suzan A. Hussain, previous reference, p. 322.
